# Renal Aspects of the Apelinergic System: Therapeutic Potential and Mechanisms

**DOI:** 10.3390/biom16020184

**Published:** 2026-01-26

**Authors:** Mulan Wang, Jun Yu, Chuanming Xu

**Affiliations:** 1 Translational Medicine Centre, Jiangxi University of Chinese Medicine, Nanchang 330004, China; 2 Center for Metabolic Disease Research, Department of Cardiovascular Sciences, Lewis Katz School of Medicine, Temple University, Philadelphia, PA 19140, USA

**Keywords:** apelinergic system, water homeostasis, kidney injury, therapeutic target

## Abstract

The Apelinergic system (AS) is a crucial endocrine system that plays a vital role in renal physiology and disease. All components of the AS are expressed throughout the kidneys in both humans and rodents. A multitude of studies have consistently shown that the AS exerts a protective effect on the kidneys across a wide spectrum of renal pathologies, encompassing acute kidney injury, chronic kidney disease, diabetic nephropathy, hypertensive kidney injury, cardiorenal syndrome, and renal cell carcinoma. Considering its pivotal role, it shows immense potential as a viable therapeutic target for renal disorders. A deeper mechanistic understanding of the AS will accelerate the rational development of novel therapeutic agents for kidney diseases. In this review, we offer a concise overview of the physiological and pathological roles of the AS in the kidney. Our focus lies on its diuretic effect and its renoprotective action against kidney injury. Enhancing the levels of peripheral Apelin or ELABELA peptides to a certain degree could potentially serve as a promising pharmacological therapeutic strategy for kidney diseases.

## 1. Introduction

The kidney is a vital organ that not only plays a crucial role in maintaining water and salt balance but also significantly contributes to cardiovascular homeostasis. Kidney diseases are major global non-communicable diseases, with a global prevalence exceeding 10%, affecting approximately 850 million people when including acute kidney injury (AKI), kidney failure, and dialysis/transplant recipients [1]. It ranks as the third fastest-growing cause of death globally, unique among non-communicable diseases in that its mortality rate increases with age [2]. Projections from global health models indicate that this condition will become the fifth leading cause of the life-years lost worldwide by 2040 [2]. Despite significant advances in understanding the pathological mechanisms underlying kidney diseases, a critical gap remains in the clinical availability of safe, effective, and disease-specific therapeutic agents or strategies. Hence, it is of utmost urgency to develop novel and potent therapeutic agents and strategies to optimize the management of kidney injury and mitigate the worldwide mortality associated with it.

The Apelinergic system (AS), an important endocrine system in the body, consists of two essential endogenous bioactive ligands, Apelin (a 77-amino-acid precursor peptide encoded by the *Apln* gene), ELABELA (ELA, also known as Toddler, a 54-amino-acid precursor peptide encoded by the *Apela* gene), and the G-protein-coupled receptor, Apelin receptor (Aplnr) [Apelin peptide jejunum (APJ), encoded by the *Aplnr* gene, was named prior to the discovery of its endogenous ligand apelin. Apelin was initially isolated from the bovine stomach by Tatemoto et al. in 1998 [3]. The designation “peptide jejunum” in APJ likely stems from the receptor’s notable expression within the jejunum.], exerting multiple biological activities in human physiologies and pathologies [4]. A variety of mature, shorter bioactive Apelin peptides (Table 1), primarily Apelin-36, Apelin-17, Apelin-13, and Apelin-12, are generated through the tissue-specific sequential proteolytic cleavage of Apelin-77, catalyzed by enzymes including aminopeptidases, serine proteases, and carboxypeptidases [5]. Specifically, the process begins with the removal of the signal peptide from Apelin-77 by signal peptidases in the endoplasmic reticulum (ER), yielding intermediates such as Apelin-51 or structurally related variants. These intermediates then undergo further cleavage in the trans-Golgi network or secretory vesicles, primarily catalyzed by proprotein convertases (PCs) such as PC1/3 and PC2. For example, PC1/3 cleaves Apelin-51 to produce Apelin-36, a major circulating isoform of apelin. Subsequent processing involves the removal of C-terminal basic residues by carboxypeptidase E. Additionally, exopeptidases such as carboxypeptidase N trim the C-terminus to generate shorter, highly active isoforms, including Apelin-17 and Apelin-13. Among them, Apelin-13 can be directly produced through the hydrolysis of Apelin-77 by proprotein convertase 3 or furin [5]. Similarly, the ELA-54 undergoes cleavage, giving rise to a mature ELA-32 peptide (a 32-amino-acid peptide). The ELA-32 peptide features two conserved di-arginine motifs, namely R^31^/R^32^ and R^42^/R^43^, which are recognized by a furin-like endopeptidase, leading to the generation of ELA-21 or ELA-11 [6]. Accumulating evidence has demonstrated that all the aforementioned mature apelin and ELA isoforms exhibit binding affinity for the APJ receptor, which couples with heterotrimeric G-proteins (Gαi, Gαq) and recruits β-arrestin to activate multiple intracellular signaling cascades, thereby regulating diverse cellular processes [7].

**Figure 1:**
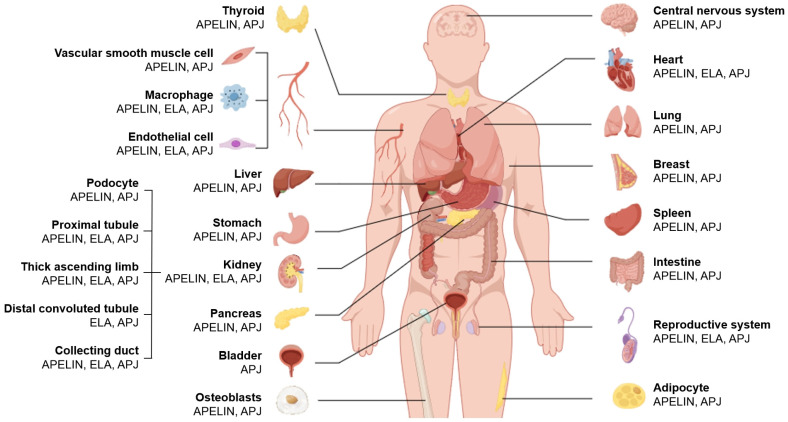
An overview of the distribution of components of the human apelinergic system.

Although several studies have shown that *Apela* mRNA expression is more widespread during the developmental stage, with high-level expression in the hearts and kidneys of embryonic rodents and limited expression only in the kidneys of adult rats and mice [17,20] and humans [21], mounting evidence indicates that the AS has a broad distribution (Figure 1) and plays a pleiotropic yet essential role in various physiological and pathological processes [22] (Table 2). Moreover, while both apelins and ELAs are detectable in circulating plasma using antibody-based enzyme-linked immunosorbent assay, their primary cellular origins have not yet been fully characterized. Concurrently, a growing body of evidence from cohort and observational studies indicates that circulating apelin and ELA levels undergo dynamic alterations across diverse pathological conditions, these alterations closely correlating with disease severity. For instance, circulating ELA concentrations are markedly diminished in patients diagnosed with heart failure [23,24,25,26], atherosclerosis [18], hypertension [27,28,29], and diabetic nephropathy (DN) [30,31], suggesting ELA may serve as a promising biomarker to facilitate early diagnosis and predict clinical outcomes of these diseases. However, due to inherent limitations in current detection methodologies, it remains challenging to accurately quantify the specific concentrations of various biologically active fragments of ELAs and apelins in the circulation.

The general landscape of the AS, including its therapeutic potential, clinical implications, and potential crosstalk with the renin–angiotensin system (RAS), has been summarized in numerous reviews [7,32,33,34,35]. The primary objective of this article is to provide a comprehensive and systematic overview of recent advances in the evolving role of the AS in renal physiology and pathophysiology, with a specific focus on its intrarenal distribution, the regulatory function of water homeostasis, and protective effects against diverse forms of kidney injury.

**Table 2:** Biological effects of the apelinergic system in multiple extrarenal organs/tissues.

Organs/Tissues	Physiological and Pathological Effects	
	Apelin/APJ	ELA/APJ
Brain	Systemic Apelin administration: cerebral injury ↓ [36]; central Apelin application: blood pressure ↑ [11,12], AVP release ↓ [10]	Systemic ELA administration: cerebral I/R injury ↓ [37,38]; central ELA application: sympathetic nerve activity ↑, blood pressure↑ [39], AVP release ↑ [40]
Heart	Systemic Apelin administration: cardiac injury ↓ [5]; central Apelin application: vascular remodeling ↑, myocardial hypertrophy ↑ [11,12]	Systemic ELA administration: cardiac injury ↓ [41]; central ELA application: vascular remodeling ↑, myocardial hypertrophy ↑ [39]
Lung	Respiratory diseases including Lung injury ↓ [42]	-
Breast	Breast tumor ↑ [43,44]	-
Spleen	Th1 cell ↓, Th2 cell ↑ [44]	-
Intestine	Colitis ↓ [45], enteric cell proliferation ↑ [46], colonic motor ↑ [47]	-
Reproductive system	Steroidogenesis ↑, folliculogenesis ↑, proliferation ↑, apoptosis ↓ [48]	Preeclampsia ↓ [49], trophoblast invasion/differentiation ↑ [50,51], syncytiotrophoblast hypoxia/reoxygenation injury ↓ [52]
Adipocyte	Adipogenesis ↓ [53], lipolysis ↓ [53], glucose uptake ↑ [54], insulin resistance ↓ [54], white Adipocytes browning ↑ [55]	-
Thyroid	Hypothyroid ↓ [56]	-
VSMC	Osteoblastic differentiation ↓ [57], vascular calcification ↓ [58]	VSMC proliferation ↓, oxidative stress ↓ [59]
Macrophage	Atherosclerosis ↓ [60,61], inflammation ↓, M1 polarization ↓, M2 polarization ↑, foam cell formation ↓ [62]	Atherosclerosis ↓ [18,63], inflammation ↓ [18,63], M1 polarization ↓, M2 polarization ↑, foam cell formation ↓ [18]
Endothelial cell	Angiogenesis ↑, endothelial injury ↓ [5]	Angiogenesis ↑ [64,65], endothelial injury ↓ [66,67]
Liver	Hepatotoxicity ↑ [45,68]	Hepatic I/R injury ↓ [69], hyperuricemia ↓ [70]
Stomach	Gastric cell proliferation ↑ [71], acid secretion ↑ [72], gastric damage ↓ [73], gastrointestinal tissue maturation ↓ [74], gastric motility ↓ [75], gastric I/R injury ↓ [76]	-
Pancreas	Insulin secretion ↓ [77], endoplasmic reticulum stress ↓ [78], pancreatitis ↓ [79], pancreatic β cell proliferation ↑ [80], endocrine pancreas damage ↓ [81]	-
Osteoblasts	Osteoclastogenesis ↑ [82], osteoporosis ↓ [83,84], Osteoblasts apoptosis ↓ [85]	Type 2 diabetes-related osteoporosis ↓, H-type angiogenesis ↑ [86]

↓ represents inhibition or downregulation, ↑ represents activation or upregulation.

## 2. Renal Distribution of the Apelinergic System

The human *APLNR* gene, located on chromosome 11 and encoding the APJ, was identified and cloned in 1993 [87]. The APJ exhibits approximately 50% homology with the type 1 angiotensin II (AngII) receptor (AT_1_R). However, it counteracts the stimulatory effect of AngII on the AT_1_R [88]. Subsequently, Apelin [3,89] and ELA [90,91], which are encoded by distinct genes (human *APLN* in chromosome X and human *APELA* in chromosome 4) and exhibit comparable biological effects, were successively identified as the endogenous ligand of the APJ. Notably, mice with Apelin [92,93] or APJ [94] deficiencies survive normally. In contrast, mice with ELA deficiency exhibited low-penetrance embryonic lethality and cardiovascular malformations [49,95]. These results indicate that maintaining normal growth and development requires ELA, but not Apelin or APJ. Evidently, despite differences among studies stemming from the limitations and detection sensitivities of selected methods, variations in species, and differences in antibodies used, accumulating evidence has demonstrated the presence of all components of the AS in the kidneys of humans and rodents (Table 3).

### 2.1. Aplnr/APJ

RT-PCR analysis demonstrated that *Aplnr* mRNA is predominantly expressed in rat glomeruli, with relatively low expression detected in renal tubules [100,107,108]. In situ hybridization histochemistry (ISHH) and RNA-Seq further confirmed that renal *Aplnr* mRNA is highly concentrated in medullary non-tubular interstitial cells, as well as endothelial and vascular smooth muscle cells of glomerular arterioles, while lower levels were detected in proximal tubules (PTs), thick ascending limbs (TALs), distal convoluted tubules (DCTs), and collecting ducts (CDs) in rodents [96,100]. Regarding the renal APJ protein, immunofluorescence staining with a rabbit monoclonal anti-APJ antibody (ab214369, Abcam, Waltham, MA, USA) demonstrated that its expression in mice is confined to the TALs in the outer medulla [16]. In contrast, a recent study by Gaydarski et al. using immunohistochemical staining with a mouse monoclonal anti-APJ immunoglobulin G (IgG) antibody (sc-517300, Santa Cruz Biotechnology, Heidelberg, Germany) demonstrated its localization in tubular epithelial cells and glomerular endothelial cells in rats [101]. In the human kidney, immunofluorescence staining with a rabbit monoclonal anti-APJ antibody (ab84296, Abcam, Cambridge, UK; or PA5-21285, ThermoFisher Scientific, Berlin, Germany) revealed that APJ protein is found in endothelial cells of intrarenal arteries and renal arterioles, podocytes in the glomerulus, as well as in the PTs, the loop of Henle, the DCTs, the juxtaglomerular apparatus, and the CDs [97,98].

### 2.2. Apln/Apelin

In rodents, ISHH and RNA-Seq analyses demonstrated that *Apln* mRNA is predominantly localized to medullary Aplnr-positive cells and is undetectable in renal tubules, vascular endothelial cells, or glomeruli [96]. Regarding the renal Apelin protein, immunohistochemical staining initially indicated the presence of Apelin protein in vascular and tubular epithelial cells, as well as in glomeruli [102]. However, through immunofluorescence staining with a rabbit monoclonal anti-Apelin antibody (ab59469, Abcam), we detected Apelin protein on the apical membrane of the principal cells in the medullary CDs in mice [16], while Nyimanu et al. reported the ubiquitously expression of APELIN protein in the human kidney, including in the endothelial cells of intrarenal arteries and renal arterioles, the podocytes within the glomerulus, the PTs, the loop of Henle, and the CDs [97].

### 2.3. Apela/ELA

In rodents, ISHH and RNAscope analysis revealed that *Apela* mRNA is consistently expressed in the renal tubular epithelial cells, with the expression strongest in the loops of Henle and CDs and very weak or absent in the glomerular [16,96]. A single-cell RNA sequencing study further confirmed the selective localization of *Apela* mRNA in the principal cells of the CDs in mice [105]. Immunofluorescence staining provided additional validation for the aforementioned findings. ELA protein is mainly expressed in renal tubules, namely the PTs, DCTs, and CDs, while only weak signals are discernible in the glomeruli in rodents [16,17,104,106]. In the human kidney, there is controversy regarding the localization of ELA protein in endothelial cells, glomeruli, and hematopoietic cells. However, immunohistochemical and immunofluorescence staining using either a rabbit monoclonal anti-[pGlu^1^]ELA-32 antibody (H-007-19, Phoenix Pharmaceuticals, Mannheim, Germany) or a custom-made antibody has shown that the ELA protein is predominantly presented in tubular epithelial cells in the PTs, the loop of Henle, DCTs, and the CDs [97,99,103].

## 3. Regulatory Potential of the Apelinergic System on Water–Salt Balance

In disorders of water metabolism, such as hyponatremia and polyuria-polydipsia syndrome, systemic apelin and arginine vasopressin (AVP) secretion exhibit significant alterations in parallel with changes in plasma osmolality [109]. Specifically, Apelin and AVP were conversely regulated by osmotic stimuli. Water deprivation (WD) or salt loading elevated plasma AVP levels and reduced plasma Apelin levels, parallel with increased plasma osmolality [10,110]. In contrast, water loading (WL) decreased plasma AVP levels and increased plasma Apelin levels, parallel with reduced plasma osmolality [110]. Interestingly, the expression of renal AS components was also significantly altered in mice subjected to WD or chronic WL treatment. Specifically, renal mRNA levels of *Apela*, *Apln*, and *Aplnr*, as well as urinary apelin excretion, were markedly reduced in WD mice but significantly elevated in WL mice [111]. Collectively, these findings demonstrate that both systemic apelin secretion and locally produced renal apelin, induced in response to osmotic stress, may contribute to the regulation of body fluid homeostasis.

**Figure 2:**
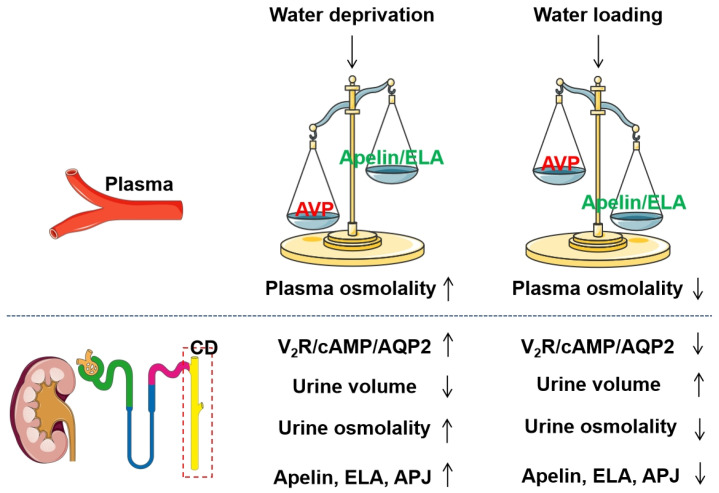
AVP and Apelin/ELA in response to osmotic stimuli. ↓ represents inhibition or downregulation, ↑ represents activation or upregulation.

Accumulating evidence has demonstrated that apelin has an antagonistic effect on the antidiuretic activity of AVP (Table 4). Apelin-13 incubation significantly antagonized AVP-stimulated aquaporin-2 (AQP2) expression by inhibiting the cAMP/PKA signaling in cultured mouse cortical CD cells [112] and primary cultured rat inner medullary CD cells [111]. Systemic administration of apelin-13 partially attenuated the increase in urine osmolality and further elevated plasma osmolality, which was associated with reduced renal AQP2 mRNA and protein expression levels [111]. Actually, the response of Apelin to WD is contrary to that of AVP [10,113] (Figure 2). Furthermore, in lactating rats, characterized by increases in both synthesis and release of AVP, intracerebroventricular administration of Apelin-17 increased diuresis, accompanied by inhibited AVP neuron activity and AVP release [10]. Intravenous injection of Apelin-17 also enhanced diuresis, accompanied by a significant reduction in urine osmolality and apical AQP2 expression in CDs [100,114]. Apelin-17 inhibited AVP/V_2_R-induced cAMP production and Ca^2+^ influx but further enhanced AVP/V_1_R-stimulated Ca^2+^ influx in the CDs [114]. Therefore, the diuretic effect of Apelin is due not just to a central action that inhibits AVP release, but also to its direct blocking of the AVP/V_2_R/AQP2 signaling in the CDs. Furthermore, similar to apelin, systemic administration of ELA-32 demonstrated superior efficacy in promoting diuresis and enhancing water intake in adult rats, as well as in stimulating ERK1/2 phosphorylation via activation of the APJ-mediated Gi signaling pathway [20]. This implies the potential of peripheral ELA in the adult kidney in regulating fluid homeostasis via activating the APJ, awaiting further validation. Of note, in contrast to Apelin, intracerebroventricular injection of ELA-32 stimulated AVP and corticotropin-releasing hormone neurons, leading to an elevation of [Ca^2+^] in the paraventricular nucleus [40]. Thus, the diuretic effect of ELA may only occur when administered systemically rather than centrally.

Notably, the clinical application of apelin peptides is limited by their short in vivo half-life [116,119]. To prolong Apelins’ in vivo half-life, Gerbier et al. generated a metabolically stable Apelin-17 analog, named LIT01-196, by conjugating a fluorocarbon chain to the N-terminal of Apelin-17 [116]. LIT01-196 not only increased the plasma half-life but also had a subnanomolar affinity for the APJ, similar to that of Apelin-17, but was superior to Apelin-17 in promoting diuresis and protecting the cardiovascular system [116]. For instance, systemic administration of LIT01-196 via intravenous or subcutaneous injection lowered blood pressure in deoxycorticosterone acetate (DOCA)-salt-induced hypertensive rats by stimulating nitric oxide (NO) synthase through the APJ [120]. Furthermore, LIT01-196 subcutaneous injection abolished the antidiuretic effect of AVP by increasing urine output, reducing urine osmolality, moderately enhancing water intake, and progressively improving hyponatremia in rats with AVP-induced hyponatremia [115]. This is probably due to the inhibition of the V_2_R/AQP2 signaling pathway through the activation of the APJ in the collecting ducts. Additionally, FC-Apelin-13, an Apelin fusion protein by conjugating the human IgG FC fragment with Apelin-13 [119], may also exhibit effects on diuresis similar to Apelin-13 and LIT01-196, which could be further clarified. Overall, activation of the APJ by Apelin peptides or their modified analogues holds promise as a potential therapeutic approach for water retention/hyponatremia.

However, the above diuretic actions of Apelin and ELA peptides may be challenged by the phenomena observed in APJ-deficient mice [94,121]. Compared with wild-type mice, although there was no difference in urine volume and osmolality, APJ-deficient mice exhibited lower water intake [94]. Although plasma AVP concentration increased comparably in both genotype mice following WD, WD or AVP-induced reduction in urine volume and increase in urine osmolality were significantly abolished in APJ-deficient mice [94,121]. In contrast, compared to wild-type mice, salt loading significantly elevated urine volume and reduced plasma AVP levels in APJ-deficient mice [121]. These results suggest that APJ-null mice lose their urinary concentrating abilities during osmotic stimulation, implying an anti-diuretic effect of endogenous Apelin and ELA peptides in vivo. This may occur through alterations in central neuroendocrine expression, dysregulation of the central AVP response to osmotic stimuli, and impairment of AVP receptor-mediated signaling pathways in the kidney [94,121]. However, the abnormal fluid homeostasis observed in APJ knockout mice also implies that APJ might not be the sole receptor for Apelin and ELA. It is plausible that additional non-APJ receptors contribute to the diuretic effects of apelin and ELA peptides, and these receptors remain to be identified. Notably, the APJ receptor exhibits high sequence homology with the AT_1_R and forms heterodimers with it, resulting in potent inhibition of AngII/AT_1_R signaling [60,122]. Although apelin was reported to induce heterodimerization between APJ and AT_1_R, causing negative allosteric regulation of AT_1_R function [122], Sun et al. demonstrated that non-activated APJ suppressed the AngII/AT_1_R signaling, an effect which was diminished by apelin-activated APJ [88]. This controversial phenomenon demonstrates the potential dual roles of the apelin/APJ signaling pathway. The abnormal fluid homeostasis observed in APJ knockout mice may be attributed to the loss of inhibition of the AngII/AT_1_R signaling.

Although intravenous injection of Apelin-17 did not influence urinary Na^+^ and K^+^ excretion in lactating rats [10,114], it significantly increased plasma Na^+^ concentrations in AVP-infused rats [115]. Several other studies imply the potential of Apelin in regulating Na^+^ balance. First, in cultured mpkCCD cells, Apelin-13 counteracts the effects of aldosterone [117]. Apelin-13 significantly reduces the amiloride-sensitive transepithelial Na^+^ current and the expression levels of epithelial sodium channel α subunit by stimulating the ERK pathway and inhibiting the serum/glucocorticoid-regulated kinase 1 and Nedd4-2 pathways [117]. Second, in WD mice, Apelin significantly suppresses renal expression of Na^+^/K^+^/2Cl^−^ cotransporter (NKCC2) [111], which is responsible for Na^+^ reabsorption in the thick ascending limb [123]. Third, in cultured murine proximal tubular cells, Apelin-13 inhibited Na^+^/H^+^ exchanger isoform 3 (NHE_3_) activity [118], which contributes to the reabsorption of 60–70% of the filtered Na^+^ [124]. Consequently, these findings imply that the AS may directly inhibit Na^+^ reabsorption in the kidneys. Nevertheless, additional research is required to validate this hypothesis and elucidate the underlying mechanisms.

## 4. Potential Roles of the Apelinergic System in Renal Pathology

The impacts of the AS on renal pathology have been extensively investigated, yielding critical insights into its multifaceted roles in the pathogenesis of acute kidney injury, chronic kidney disease, diabetic nephropathy, hypertensive kidney injury, cardiorenal syndrome, and renal cell carcinoma. Comprehensive analyses of diverse experimental models, therapeutic intervention strategies, underlying molecular mechanisms, and clinical outcomes associated with AS modulation have unveiled its substantial therapeutic potential in mitigating these renal injury conditions. These discoveries carry far-reaching implications, opening novel avenues for targeted therapeutic interventions and advancing our understanding of the intricate molecular underpinnings of kidney injuries.

### 4.1. Acute Kidney Injury

In a clinical setting, ischemia–reperfusion (I/R) injury is the most frequent cause of AKI [125], which serves as a crucial factor influencing morbidity and mortality during the perioperative period [126]. Patients with I/R-induced AKI are predisposed to a heightened risk of developing chronic kidney disease (CKD) [127]. In I/R-induced AKI mice, the renal levels of APJ were higher, but the renal levels of Apelin were lower than in sham mice [118], indicating a possible Apelin-APJ feedback loop in the tubules of I/R mice, in which the reduced Apelin levels may be a consequence of kidney injury, to limit the renal Apelin/APJ signaling. A series of reports suggests that Apelin-13 can effectively alleviate I/R-induced AKI in rodents [128,129,130,131,132,133], potentially providing promising therapeutic options for the clinical management of I/R-induced AKI. Specificity, Apelin-13 significantly mitigated renal I/R injury and inhibited apoptosis by restoring lysosomal membrane permeability, augmenting lysosomal biogenesis, and enhancing autophagy in mice [129,130]. In rats with I/R-induced AKI, the exogenous administration of Apelin-13 effectively attenuated the deleterious effects on renal function and histological architecture by ameliorating oxidative stress through increased antioxidant enzyme activity and Nrf2 expression [132,133]. Furthermore, subsequent investigations have confirmed that Apelin-13 exerts a renoprotective effect against I/R-induced AKI by activating the APJ receptor pathway, which inhibits renal cell apoptosis, oxidative stress, inflammation, and interstitial fibrosis in rats [128,131,134]. Thus, these reports unanimously indicate the renoprotective effect of Apelin-13 in a dose of 5–30 μg/kg/day against I/R-induced AKI. However, a recent study by Lopes-Gonçalves et al. demonstrated that a high dose of Apelin-13 (200 μg/kg/day) did not improve renal I/R injury in mice. Notably, this high-dose treatment also suppressed tubular epithelial cell proliferation and impaired the adaptive repair response to renal I/R injury [118]. Therefore, the alleviating effect of Apelin-13 on I/R-induced AKI may have a dose-dependent biphasic effect (Table 5).

**Figure 3:**
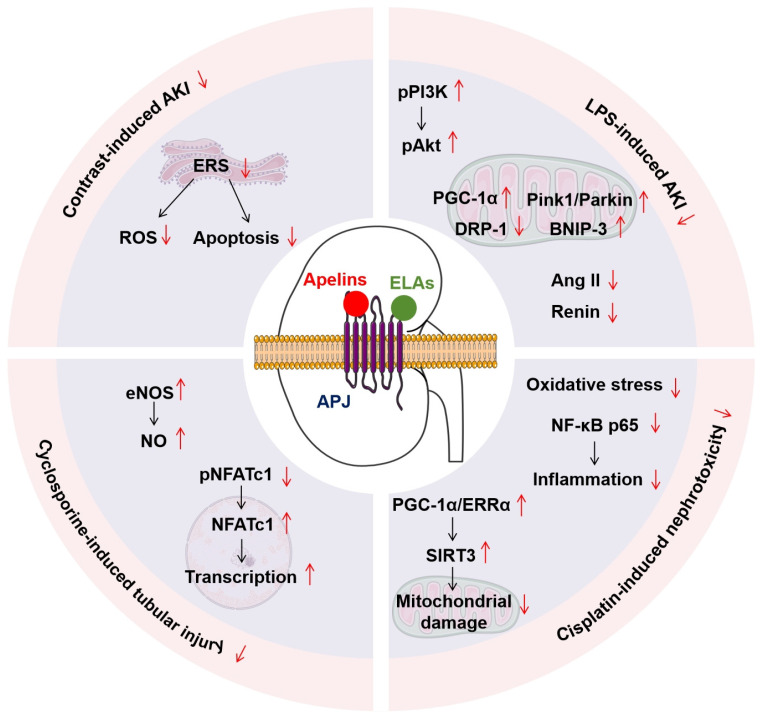
The cellular antitoxic effects of the renal apelinergic system. The red ↓ represents inhibition or downregulation, the red ↑ represents activation or upregulation, the black ↓ represents signal transmission direction.

AKI can also be triggered by nephrotoxicity of several toxic substances and specific drugs, along with sepsis, such as contrast media, cisplatin, cyclosporine, and lipopolysaccharide [146]. Interestingly, Apelin-13 has a similar antagonistic effect on AKI caused by these mentioned drugs (Figure 3). For example, emerging hypotheses suggest that Apelin-13 potently attenuates contrast-induced AKI through its dual antioxidant and cytoprotective actions [147]. In fact, contrast media significantly decreased the expression levels of renal Apelin protein. The application of exogenous Apelin-13 can improve contrast-induced AKI by reducing oxidative stress and apoptosis, at least in part by alleviating ER stress in renal tubular epithelial cells [135]. Apelin-13 administration significantly mitigates cisplatin-induced nephrotoxicity by reducing oxidative stress, inhibiting NF-κB p65-mediated inflammation [136], and protecting renal tubular mitochondria through the activation of the PGC-1α/ERRα/SIRT3 signaling [137]. Apelin-13 also antagonized cyclosporine-induced AKI in rats by activating NO and the nuclear factor of activated T-cell cytoplasmic 1 (NFATc1) pathway [138]. Moreover, Apelin-13 significantly ameliorated lipopolysaccharide-induced AKI in rats by improving mitochondrial quality and inhibiting the intrarenal RAS [139].

In I/R-induced AKI mice, renal *Apela* mRNA levels were also significantly reduced [17]. Studies have demonstrated that both ELA-32 and ELA-11 exert significant renoprotective effects in a murine model of renal I/R injury, as evidenced by their ability to alleviate renal fibrosis, suppress inflammatory responses, inhibit tubular epithelial cell apoptosis, and attenuate DNA damage responses. Notably, these peptides also mitigate renal tubular lesions and improve markers of renal dysfunction [17]. Contrary to initial expectations that these peptides would confer significant therapeutic benefits against I/R-induced AKI, both ELA-32 and ELA-11 exhibit a short in vivo half-life [119,148] with relatively low binding affinity for the APJ receptor, which substantially limits their translational potential for clinical applications. To overcome these limitations, researchers have explored structural modifications of these peptides using various strategies, including conjugation with palmitic acid (Pal), polyethylene glycol (PEG) [140], or the Fc fragment of immunoglobulin G (IgG) [141,142]. Notably, PEGylated and acylated ELA-11 analogues exhibit enhanced in vivo stability and APJ-binding affinity, and demonstrate superior renoprotective efficacy compared to native ELA-11 in a murine model of renal I/R injury [140]. Similarly, Fc-fused ELA-21 (Fc-ELA-21) significantly prolongs in vivo half-life in mice following administration, and functionally outperforms native ELA-21 in mitigating LPS- or I/R-induced AKI through activation of the PI3K/Akt signaling pathway [141,142]. These results consistently indicate that exogenous ELA peptides, including ELA-32, ELA-21, and ELA-11, could serve as effective therapeutic candidates for the treatment of AKI. Furthermore, Xiong and colleagues recently reported that renal tubule-specific ELA deletion aggravated pathological renal injury and the subsequent AKI-CKD transition in mice with I/R-induced AKI [104]. This exacerbation is driven by the aggravation of renal microvascular injury, which is mediated by APJ receptor-dependent activation of the arginine-metabolizing enzyme arginase 2 and the inhibition of prostaglandin E_2_ synthesis [104]. Thus, ELA peptides may act as antagonists against AKI via the APJ. However, this notion necessitates further validation, particularly through the use of a renal tubular-specific APJ deletion model.

Nevertheless, Apelin and ELA peptides could serve as effective agents for the therapy of AKI, thereby significantly impeding the progression of CKD.

### 4.2. Chronic Kidney Disease

Renal fibrosis, which serves as the shared pathological feature of diverse CKDs, including autosomal dominant polycystic kidney disease (ADPKD), is marked by the excessive deposition of extracellular matrix (ECM) in kidney tissues and progressively develops to end-stage renal failure [149]. In response to unilateral ureteral obstruction (UUO), although plasma Apelin levels were reduced in mice [143], the renal Apelin and APJ expression showed a compensatory significant increase [143,144]. Apelin-13 significantly ameliorated renal interstitial fibrosis in the UUO mice [143,144] by antagonizing the TGF-β1/SMAD-stimulated ECM [150,151] and activating the Akt/eNOS pathway [144]. Similarly, ELA-32 also attenuated ECM by inhibiting the TGF-β/SMAD, ERK1/2, and AKT pathways [152], implying the potential of ELA to antagonize renal interstitial fibrosis. This finding is supported by experimental observations that ELA-13 potently abrogates UUO-induced renal fibrosis in mice through inhibiting the TGF-β/SMAD and ERK1/2 signaling pathways [145]. Collectively, these studies highlight the therapeutic potential of Apelin and ELA peptides for CKD by targeting renal fibrosis, as summarized in Table 5.

Some studies have clinically investigated the association between Apelin/Elabela ELA and CKD. Nyimanu et al. demonstrated that plasma Apelin levels (but not ELA levels) were elevated in patients with CKD and were negatively correlated with kidney function [97]. In contrast, other studies have reported conflicting results. Lu et al. found that serum ELA levels (but not apelin levels) gradually and significantly decreased with declining estimated glomerular filtration rate (eGFR) in CKD patients [153]. Rahmati et al. [154] and Coskun et al. [155] observed no significant difference in serum Apelin levels between CKD patients and healthy controls, suggesting that plasma Apelin levels may not serve as a reliable biomarker for CKD. The underlying reasons for discrepancies in circulating Apelin or ELA levels among patients with CKD remain elusive, but they may be attributed to differences in patient cohorts (e.g., variable CKD stages, comorbid conditions) or methodological variations across studies (e.g., sample types, detection assays employed). Another plausible explanation is that complex interactions between comorbidities and disease progression may obscure the potential association between serum Apelin levels and the progression of CKD. Chapman et al. conducted a randomized, double-blind, placebo-controlled, crossover study to explore the therapeutic potential of Apelin-13 in CKD patients (NCT03956576). The study preliminarily demonstrated that [Pyr^1^]Apelin-13 confers short-term acute cardiovascular and renal benefits in CKD patients, including reduced mean arterial pressure and systemic vascular resistance, increased cardiac index, renal blood flow, natriuresis, and free water clearance, and reduced GFR, filtration fraction, and proteinuria [15]. While the chronic effects of [Pyr^1^]Apelin-13 were not evaluated in this study, their potential to improve patient outcomes appears promising. Although it is theoretically speculated that ELA peptides may exert similar beneficial effects in CKD patients as Apelin-13, there is currently a dearth of research in this field. Therefore, clinical studies to explore the therapeutic potential of ELA peptides in CKD, along with clinical trials regarding the long-acting application of Apelin or ELA peptides, are still needed.

In particular, when compared with healthy controls, serum Apelin levels were significantly lower in patients with ADPKD [156,157,158]. These levels exhibited an inverse association with TGF-β1 and a positive correlation with eGFR [157]. Furthermore, serum Apelin levels exhibit robust diagnostic performance in identifying disease progression in ADPKD patients [158]. Notably, there is still a lack of relevant reports on the impact of Apelin or ELA on ADPKD pathogenesis. However, considering the well-established pathogenic role of AVP/V_2_R signaling in driving ADPKD progression [159,160], we hypothesize that Apelin or ELA may exert renoprotective effects in ADPKD. This speculation is supported by their previously documented antagonistic actions on the AVP/V_2_R signaling pathway, as discussed earlier in this review. This is of greater interest to clarify in the future.

### 4.3. Diabetic Nephropathy

DN is a severe microvascular complication that may develop into severe end-stage renal failure [161]. The primary pathological feature of this complication is TGF-β1-driven renal fibrosis [162]. Serum Apelin-13 levels were notably elevated in DN patients and negatively correlated with serum TGF-β1 levels [163]. In patients with type 2 diabetes, serum Apelin levels were significantly increased [164] and positively associated with urinary albumin excretion [165]. These suggest the potential of serum Apelin-13 as an indicator for renal fibrosis or destruction in DN patients. Notably, controversy remains regarding the effects of exogenous Apelin-13 on diabetic nephropathy (DN). A series of studies by the Zeng group has demonstrated that elevated Apelin-13 levels promote DN progression through well-characterized cellular and molecular mechanisms [165,166,167]. Specifically, Apelin-13 induced podocyte apoptosis, renal functional impairment, and foot process injuries by suppressing podocyte autophagy in spontaneously type 2 diabetic KKAy mice [166]. It caused podocyte dysfunction by initiating ER stress through the reduction in proteasome activities [167]. Furthermore, in glomerular endothelial cells, Apelin-13 enhanced permeability by upregulating the expression of VEGFR2 and Tie2 [165]. The above effects thereby facilitate the advancement of DN. However, subsequent reports from the same group showed that Apelin-13 plays a protective role in streptozotocin-induced diabetic mice [168,169] and spontaneously type 2 diabetic KKAy mice [170] via distinct mechanisms. In podocytes, Apelin-13 inhibited EMT to reduce glomerular fibrosis by suppressing the β5i/TGF-β1/SMAD pathway [170]. In addition, in glomerular endothelial cells, Apelin-13 reduced glomerular basement membrane thickening in diabetic mice by preventing extracellular matrix synthesis via stimulating the SIRT3/KLF15 pathway [168] or alleviating endothelial-to-mesenchymal transition via increasing large conductance calcium-activated potassium channels to inhibit the Wnt/β-Catenin pathway [169]. In fact, the protective effect of Apelin-13 against DN has been confirmed by multiple research groups. Day et al. found that the administration of Apelin-13 had distinct effects depending on the treatment duration in mice with type 1 diabetes. A short-term treatment with Apelin-13 significantly reduced kidney and glomerular hypertrophy as well as renal inflammation. Meanwhile, a long-term treatment improved albuminuria. These beneficial effects were achieved by stimulating the antioxidant catalase [171]. Gao et al. reported that Apelin-13 mitigated DN by enhancing the biosynthesis of NO and suppressing renal fibrosis in streptozocin-injected rats [172]. Moreover, Chen et al. conducted experiments in spontaneous type 1 diabetic Akita mice, in which Apelin-13 attenuated a wide range of diabetes-induced renal manifestations, including a decrease in the glomerular filtration rate, the presence of proteinuria, glomerular enlargement, mesangial proliferation, and renal inflammatory responses, by inhibiting histone acetylation [173]. All these reports uniformly imply that Apelin-13 may have an impact on the progression of DN. Yet, it is of great importance that the exact therapeutic potential of Apelin-13 for DN requires further validation through well-designed clinical studies.

Compared to Apelin, the impact of ELA on DN is more evident. In patients with type 2 diabetes mellitus, serum ELA levels progressively decrease with the progression from normoalbuminuria to microalbuminuria and macroalbuminuria, and are positively correlated with eGFR [30,31]. These findings suggest that ELA may hold diagnostic and therapeutic potential for patients with DN. Renal ELA mRNA and protein levels were significantly reduced in db/db DN mice [66,174], which may be involved in the development of DN. Administration of ELA-21 attenuated renal inflammation and fibrosis, and ameliorated podocyte apoptosis by activating the PI3K/Akt/mTOR signaling pathway, thereby improving renal pathological changes and kidney dysfunction in streptozotocin-induced type 1 diabetic mice [175]. Additionally, ELA-21 exerted renoprotective effects in db/db mice by activating renal tubular autophagy, which attenuated renal pathological damage and interstitial fibrosis [174]. These pharmacological studies preliminarily indicate the antagonistic effect of ELA peptides on DN, which is further confirmed in transgenic mice. ELA deficiency in mice notably expedited the diabetic glomerular injury induced by streptozotocin, which was manifested by aggravated glomerular morphological damage, an increase in serum creatinine and blood urea nitrogen levels, and a rise in urinary albumin excretion [66]. Conversely, intrarenal transfection of ELA lentivirus to achieve ELA overexpression in db/db mice remarkably lowered serum creatinine and blood urea nitrogen levels, diminished urinary albumin excretion, and mitigated glomerular endothelial injury [66]. The beneficial effects of ELA overexpression were mediated by the activation of the APJ receptor, which subsequently modulated the AMPK/NLRP3 signaling pathway to exert renoprotective effects [66]. Therefore, pharmacological targeting of ELA may be considered as a novel and potentially effective therapeutic strategy for DN (Table 6).

### 4.4. Hypertensive Kidney Injury

In patients with hypertension, the serum Apelin levels were significantly decreased and showed a negative correlation with the risk of hypertension [176,177,178]. The expression of Apelin and APJ in kidney tissue was significantly reduced in L-NAME-induced hypertensive rats [179]. However, in spontaneously hypertensive rats, the expression of the APJ was significantly upregulated in both glomeruli and renal tubules compared to normotensive control rats. Moreover, it was positively correlated with the glomerular sclerosis index and the tubulointerstitial damage index [101,180], respectively. Thus, the level of Apelin and Apelin receptor may be related to specific hypertension models. Indeed, the effect of Apelin on blood pressure also exhibits a dependence on hypertension models. For example, in hypertension induced by L-NAME or asymmetric dimethylarginine, which is associated with endothelial damage, intraperitoneal injection of Apelin-13 exacerbated the hypertension [181,182]. However, in rats with two-kidney-one-clip-induced chronic renovascular hypertension [183,184,185,186] or spontaneously hypertensive rats [187], intraperitoneal injection of Apelin-13 exhibited antihypertensive effects. Moreover, systemic administration of LIT01-196, a metabolically stable Apelin-17 analog, via intravenous or subcutaneous injection, decreased blood pressure in DOCA-salt-induced hypertensive rats [120]. More importantly, microinjection of apelin-13 into the paraventricular nucleus of spontaneously hypertensive rats resulted in elevated blood pressure, which was mediated by increased sympathetic activity and stimulated release of AVP [11,12]. Thus, as concluded by Mohammadi et al., the impact of Apelin on hypertension is closely related to the route of administration. Specifically, peripheral administration of Apelin reduces blood pressure, whereas central administration of Apelin exacerbates hypertension [188]. Moreover, subcutaneous injection of apelin-13 significantly improved blood pressure, renal function, and renal structural damage in a rat model of L-NAME-induced preeclampsia. These beneficial effects were attributed to increased nitric oxide (NO) production, reduced endothelin-1 (ET-1) bioavailability, and inhibition of reactive oxygen species (ROS) generation, lipid peroxidation, and inflammatory responses in both the maternal circulation and renal tissues [189]. Therefore, systemic administration of Apelin may exert a certain antagonistic effect against hypertensive kidney injury.

Interestingly, similar to Apelin, the effect of ELA on blood pressure also exhibits a dependence on the route of administration [6,41]. Specifically, peripheral administration of ELA exhibits antihypertensive and renoprotective effects [16,59,106,190]. In contrast, central administration of ELA raises blood pressure [39]. In hypertensive patients, serum ELA levels showed a progressive decline as blood pressure continued to rise [27,28,29]. These levels were negatively correlated with serum creatinine and blood urea nitrogen, while being positively correlated with the eGFR, suggesting that ELA has the potential to serve as a marker for hypertension-related kidney damage [27]. Moreover, the expression of renal *Apela*, *Apln*, and *Aplnr* mRNA in high-salt-fed Dahl salt-sensitive (DSS) rats [16], as well as renal *Apela* mRNA and ELA protein levels in DOCA/salt-treated rats [190], were significantly reduced. These results suggest that the decrease in renal ELA may promote hypertensive kidney injury. To support this view, in high-salt-fed DSS rats, either subcutaneous infusion of exogenous ELA-32 peptide [16] or intravenous injection of adeno-associated virus serotype 9 (AAV9) for *Apela* gene delivery [106], and in DOCA/salt-treated rats, intrarenal ELA-32 overexpression by intrarenal transfecting the *Apela* overexpression plasmid pcDNA3.1-Apela [190], were found to significantly inhibit renal inflammation, fibrosis, and kidney injury. This inhibition may be achieved by suppressing the intrarenal RAS [16] and the NADPH oxidase/ROS/NLRP3 pathway [190]. Moreover, in spontaneously hypertensive rats fed a high-salt diet, ELA-32 infusion was superior to Apelin-13 in reducing cardiovascular and renal dysfunctions, fibrosis, and hypertrophy. This superiority is likely attributed to ELA-32′s higher affinity for the APJ, its better resistance to degradation by RAS enzymes, and its ability to attenuate the overactivation of the cardiac and intrarenal RAS [191]. Therefore, these results suggest that peripheral ELA administration has nephroprotective effects against hypertensive kidney injury (Table 7).

### 4.5. Cardiorenal Syndrome

Accumulating evidence has established a close relationship between Apelin/ELA and various heart diseases, highlighting the therapeutic potential of peripheral Apelin/ELA administration for these conditions [22,41]. Notably, due to the heterogeneity in disease severity, clinical complications, and patient demographics across different cohorts, inconsistent changes in circulating apelin or ELA levels have been reported in patients with cardiovascular diseases. For example, some studies have reported elevated plasma Apelin levels in heart failure patients with preserved or mid-range ejection fraction (HFpEF or HFmrEF) [26,198], while other reports have demonstrated a significant decrease in plasma Apelin levels in heart failure patients with reduced ejection fraction (HFrEF) [199,200,201,202] and atrial fibrillation patients [203]. For another instance, plasma ELA levels were elevated in patients with coronary heart disease [204,205,206] and HFrEF patients combined with well-preserved renal function [207], but reduced in patients with congenital heart disease [23], atrial fibrillation patients [24], hypertensive patients with HFmrEF [25], and HFpEF patients [26]. Nonetheless, the application of Apelin [192,193,194] or ELA [41] peripherally exhibited a significant cardio-protective action against pathological myocardial injury.

Once patients with heart failure are diagnosed with renal dysfunction, this condition is referred to as cardiorenal syndrome (CRS) [208]. Notably, in patients with CRS secondary to HFrEF, who exhibit concurrent deterioration of both renal and cardiac function, serum ELA levels were found to positively correlate with eGFR [207]. This finding underscores the potential role of ELA in maintaining renal function in patients with CRS. Studies by the Wang group have demonstrated that, in mice with CRS triggered by the ligation of the left anterior descending coronary artery, subcutaneous infusion of ELA-32 can significantly improve cardiac and renal function by inhibiting the NF-κB signalling pathway via the APJ [195,196]. Peripheral infusion of exogenous ELA-32 or Apelin-13 has also shown beneficial effects on cardio-renal function in other animal models. In septic rats induced by cecal ligation and puncture, ELA-32 infusion was superior to Apelin-13 in alleviating myocardial and renal dysfunction by distinctively inhibiting the release of pituitary vasopressin [197]. Thus, ELA peptides may be ideal therapeutic agents for CRS (Table 7).

### 4.6. Renal Cell Carcinoma

Accumulating evidence has established the critical role of the AS in both cancer diagnosis and therapeutic response [209,210]. Indeed, the APJ has been acknowledged as an indispensable factor for efficient cancer immunotherapy by interacting with Janus Kinase 1, modulating interferon-γ responses in tumours, and influencing the effector function of CD8^+^ T cells. Evidently, the absence of APJ is correlated with the inefficacy of cancer immunotherapy [211]. While the GSE6344 dataset suggested upregulation of APLN mRNA levels in clear cell renal cell carcinoma (ccRCC) [212], subsequent RT-qPCR analyses revealed no statistically significant difference in APLN mRNA expression between ccRCC tissues and adjacent normal controls [98,212]. The expression levels of *APLN* mRNA showed only a weak correlation with tumor histological grade [98]. Conversely, the levels of APJ protein were negatively correlated with the aggressiveness of ccRCC and the levels of programmed cell death ligand 1 by tumour cells in ccRCC [98]. Furthermore, ELA expression differs significantly between benign and malignant renal tumors [103]. Specifically, relative to control kidney tissue, ELA protein levels were markedly elevated in renal oncocytoma (a benign tumor) yet reduced in chromophobe RCC, papillary RCC, and ccRCC, the three most common malignant subtypes. Notably, within ccRCC, ELA levels were higher in Fuhrman Grade 4 (the most aggressive histologic grade) compared to Grades 1–3. Collectively, these findings imply the critical roles of the Apelin-ELA/APJ signaling pathway in the pathogenesis and progression of RCC. Notably, while Apelin is well-documented to drive cancer initiation and maintenance [210,213], the effects of ELA on tumor biology appear to be tumor-type dependent. For example, exogenous ELA-32 promoted the progression of ovarian clear cell carcinoma (OCCC) through APJ-independent inhibition of p53 expression, whereas silencing ELA expression attenuated OCCC tumorigenesis [214]. In contrast, ELA showed a tumor suppressor function in RCC through activating the mammalian target of rapamycin complex 1 (mTORC1) [99]. Overall, while the precise role of Apelin in RCC remains to be fully elucidated, the protective effect of ELA against renal malignancies has been experimentally validated. Due to the similar biological effects of Apelin and ELA, Apelin may have an antagonistic effect on RCC similar to that of ELA, which awaits further verification.

## 5. Future Prospective

Despite significant progress in elucidating the role of the AS in various disease states, direct evidence linking the AS modulation to patient survival outcomes or the long-term therapeutic efficacy of chronic apelin or ELA peptides administration remains elusive. Furthermore, several critical knowledge gaps persist in this field. First, clinical investigations into the renal physiological and pathological effects of apelin or ELA peptides are still relatively limited. Current evidence suggests that the therapeutic application of apelin or ELA peptides in kidney diseases requires systemic rather than central administration, an approach that may elicit unintended off-target effects. A notable example is the promotion of pathological angiogenesis [41,215], a critical consideration particularly for patients with comorbidities. Furthermore, the dosage of these peptides must be carefully optimized, as high doses have been associated with adverse effects [118,216].

Second, the short in vivo half-life of Apelinergic peptides [119,148,217] has posed significant challenges in accurately quantifying their levels in vivo and implementing them in clinical practice. In light of these circumstances, continuous efforts are being made to explore numerous small-molecule, peptide-based agonists, antagonists, and allosteric modulators of APJ [218], along with more stable peptides and non-peptide derivatives of Apelin and ELA. For instance, to enhance pharmacological stability, several strategies have been employed. These include N-PEGylation and N-palmitoylation of native peptides [140], amino acid substitution and fatty acid modification of native peptides [219,220], the fusion of Apelin-13 or ELA-21 to an IgG FC fragment [119,148] or a fluorocarbon chain [116], and the encapsulation of [Pyr^1^]Apelin-13 in PEGylated liposomal nanocarriers [221]. Furthermore, small-molecule APJ agonists such as MM07 [217], CMF-019 [222], and AMG986 [223], as well as antibody agonists against APJ [224], were developed successively. All these aforementioned efforts would increase the potential for the pharmacological utility of Apelinergic peptides.

Third, many theoretical issues still need clarification. For example, it is essential to determine the renal distributions, as well as the physiological and pathological roles, of Apelinergic peptide isoforms. For another example, the principal signaling pathways within the renal AS that mediate its renal effects remain to be fully elucidated, highlighting a critical gap in our understanding of AS’s physiological and pathophysiological roles in renal function. Moreover, further clarification is also needed on whether Apelin and ELA peptides exert their renal effects through renal APJ. Indeed, the regulatory impact of APJ deficiency on water balance is comparable to that exerted by Apelin and ELA peptides [94,121]. When Aplnr expression was knocked down using small interfering RNA in cultured NRK-52E renal tubular epithelial cells, this intervention did not attenuate the cytoprotective effects of ELA-11 and ELA-32 against hypoxia–reperfusion injury [17]. Collectively, these findings suggest the potential existence of non-APJ receptors in the kidney that mediate ELA peptide signaling, which requires further experimental validation through complementary approaches such as receptor binding assays or CRISPR-Cas9-mediated gene knockout studies.

## 6. Conclusions

**Figure 4:**
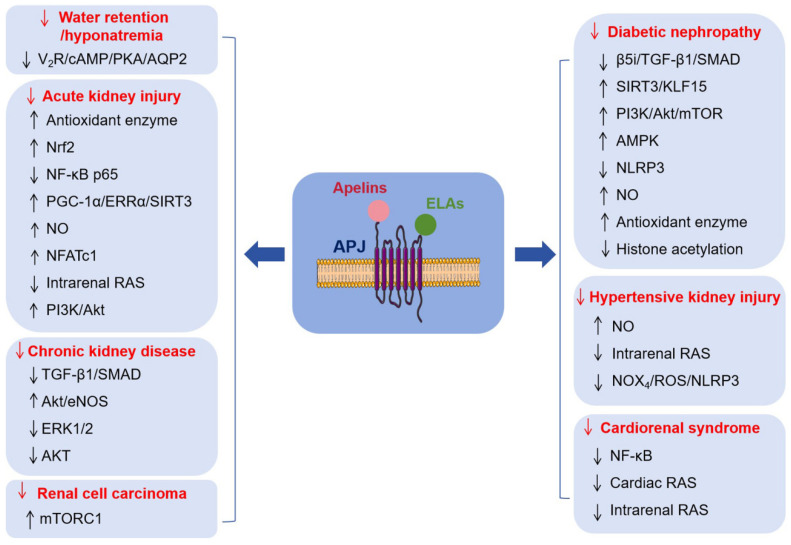
Specific functions of the Apelinergic system in various renal pathologies and the potential underlying molecular mechanisms. The Apelinergic system exerts a potential renoprotective action antagonizing acute kidney injury, chronic kidney disease, diabetic nephropathy, hypertensive kidney injury, cardiorenal syndrome, and renal cell carcinoma. ↓ represents inhibition or downregulation, ↑ represents activation or upregulation.

In summary, targeting the AS represents a highly promising therapeutic strategy, primarily due to its potential to confer beneficial effects on renal function. These effects encompass the regulation of water–salt homeostasis and the amelioration of diverse renal pathologies, including AKI, CKD, DN, hypertensive kidney injury, CRS, and RCC (Figure 4). The AS undoubtedly warrants in-depth investigation, as it holds significant promise as a therapeutic target capable of providing renal protection and potentially improving the prognosis of patients with kidney diseases.

## Figures and Tables

**Table 1:** The active fragments of human Apelins and ELAs.

Active Fragments	Amino Acid Sequence	Partial Representative Biological Activities
Apelin-36	LVQPRGSRNGPGPWQGGRRKFRRQRPRLSHKGPMPF	Trophoblast cell injury ↓ [8], acute lung injury ↓ [9]
Apelin-17	KFRRQRPRLSHKGPMPF	Plasma AVP levels ↓ [10], diuresis ↑ [10]
Apelin-13	QRPRLSHKGPMPF	Central application: vascular remodeling ↑, myocardial hypertrophy ↑, blood pressure ↑ [11,12]
Apelin-12	RPRLSHKGPMPF	Neonatal lung injury ↓ [13], cellular senescence ↓ [14]
[Pyr^1^]apelin-13	(Pyr)RPRLSHKGPMPF	Short-term cardiovascular and renal benefits in CKD patients [15]
ELA-32	QRPVNLTMRRKLRKHNCLQRRCMPLHSRVPFP	Systemic application: hypertension and kidney injury ↓ [16,17]
ELA-21	LRKHNCLQRRCMPLHSRVPFP	Atherosclerosis ↓ [18]
ELA-14	QRRCMPLHSRVPFP	Myocardial fibrosis ↓ [19]
ELA-11	CMPLHSRVPFP	Renal I/R injury ↓ [17]

↓ represents inhibition or downregulation, ↑ represents activation or upregulation.

**Table 3:** The cellular distribution of components of the renal apelinergic system.

	Human			Rodent (Mouse or Rat)		
	*APLNR*/APJ	*APLN*/APELIN	*APELA*/ELA	*Aplnr*/APJ	*Apln*/APELN	*Apela*/ELA
Interstitial cell	-	-	-	m [96]	m [96]	-
Podocyte	p [97,98]	p [97]	-	-	-	-
Endothelial cell	p [97,98,99]	p [97]	-	m [100], p [101]	p [102]	-
vascular smooth muscle cell	p [97,98]	-	-	m [100]	p [102]	-
Juxtaglomerular apparatus	p [97,98]	-	-	m [100]	-	-
Proximal tubule	p [97,98,99]	p [97]	p [97,99,103]	m [100], p [101]	p [102]	m [96], p [17,104]
Thick ascending limb	p [97,98,99]	p [97]	p [97,99]	m [100], p [16,101]	p [102]	m [96], p [17,104]
Distal convoluted tubule	p [97,98,99]	-	p [97,99,103]	m [100], p [101]	p [102]	m [96], p [17,104]
Collecting duct	p [97,98,99]	p [97]	p [97,99,103]	m [100], p [101]	p [16]	m [16,96,105], p [16,17,104,106]

p, protein; m, mRNA.

**Table 4:** Effects of exogenous Apelins or ELAs on water–salt balance.

Peptides/Analogs	Experimental Models	Dose and Route	Outcomes	Associated Molecular Mechanism	Refs
Apelin-13	AVP-treated mpkCCD cells	-	*Aqp2* mRNA ↓, AQP2 ↓, AQP2-pS269 ↓	cAMP production ↓	[112]
Apelin-13	AVP-treated IMCD cells	-	AQP2 ↓	cAMP/PKA/sPRR signaling ↓	[111]
Apelin-13	WD mice	100 μg/kg/day, i.p.	Urine osmolality ↓, plasma osmolality ↑	Renal AQP2 expression ↓	[111]
Apelin-17	Lactating rats	2 μg, i.c.v.	Plasma AVP levels ↓, diuresis ↑	The phasic electrical activity of AVP neurons ↓	[10]
Apelin-17	Lactating rats	0.48, 1.90, 3.80 nmol, i.v.	Diuresis ↑	Vasorelaxation of efferent and afferent arterioles ↓, NO ↑, intracellular Ca^2+^ ↓	[100]
Apelin-17	Lactating rats	3.8 nmol/2 h, i.v.	Diuresis ↑, urine osmolality ↓	AVP/V2R-induced cAMP production and Ca^2+^ influx in the CDs ↓	[114]
ELA-32	Adult rats	2, 5, 10, 20 nmol, i.v.	Diuresis ↑, water intake ↑	APJ-mediated Gi signaling ↑	[20]
ELA-32	Adult mice	100, 300 pmol, i.c.v.	Food intake ↑, AVP and corticotropin-releasing hormone neurons ↑, intracellular Ca^2+^ ↑	-	[40]
LIT01-196 (Apelin-17 analog)	Rats with AVP-induced hyponatremia	900 nmol/kg, s.c.	Diuresis ↑, urinary osmolality ↓, plasma Na^+^ concentration ↑	-	[115]
LIT01-196	WD mice	0.0001–1 mg, i.c.v.	AVP release ↓	-	[116]
LIT01-196	Normotensive rats	0.0001–1 mg, i.v.	Diuresis ↑, arterial blood pressure ↓	-	[116]
Apelin-13	mpkCCD cells	-	Amiloride-sensitive transepithelial Na^+^ current ↓, α-ENaC expression ↓	ERK pathway ↑, SGK1 and Nedd4-2 signalings ↓	[117]
Apelin-13	WD mice	100 μg/kg/day,i.p.	NKCC2 expression ↓	-	[111]
Apelin-13	Murine proximal tubular cells (TKPTS)	-	NHE_3_ activity ↓	-	[118]

↓ represents inhibition or downregulation, ↑ represents activation or upregulation.

**Table 5:** Effects of exogenous Apelins or ELAs on acute kidney injury and chronic kidney disease.

Peptides/Analogs/Antagonists	Experimental Models	Dose and Route	Outcomes	Associated Molecular Mechanism	Refs
**Acute kidney injury**					
Apelin-13	Mice with renal I/R injury	200 μg/kg/day, i.p.	Podocyte injury ↓	Podocyte autophagy ↑, m-TOR phosphorylation ↓, podocyte apoptosis ↓	[129]
Apelin-13	Male rats with renal I/R injury	5 μg/kg/day, s.c.	Tubular lesions ↓, cell apoptosis ↓, inflammation ↓	TGF-β1 ↓	[131]
Apelin-13	Male rats with renal I/R injury	1, 10, 100 μg/kg, i.p.	Serum total protein ↓, albumin ↓, TAS ↓, BUN ↑, CRE ↑, TNF-α ↑	Antioxidant enzyme activity ↑	[133]
Apelin lentivirus	High-fat diet-fed rats with renal I/R injury	t.v.i.	Serum BUN ↓, CRE ↓, cell apoptosis ↓, inflammation ↓,ROS ↓	Nrf2 signaling ↑	[132]
Apelin-13	Mice with renal I/R injury	30 μg/kg/day, i.p.	Serum BUN ↓, CRE ↓, cell apoptosis ↓, M2 macrophages ↑, M1 macrophages ↓	Lysosomal membrane permeability ↑, lysosomal biogenesis ↑, autophagic flux ↑	[130]
Apelin-13	Rat kidney transplantation with renal I/R injury	100 μg/kg/day, i.p.	Serum BUN ↓, CRE ↓, tubular architectural disruption ↓, cell apoptosis ↓, inflammation ↓, M1 macrophages ↓	NF-κB ↓, KLF2 ↑	[134]
Apelin-13	Mice with renal I/R injury	200 μg/kg/day, i.p.	Tubular injury ↕, CRE clearance ↕, plasma urea level ↕	Tubular proliferation ↓, the adaptive response to renal I/R injury ↓	[118]
Apelin-13	Contrast-induced AKI rats	10 , 100 nM/kg, t.v.i.	ROS ↓, apoptosis ↓	ERS ↓	[135]
Apelin-13	Cisplatin-induced AKI rats	20 nmol kg/day, i.p.	Kidney damage ↓	Oxidative stress ↓, NF-κB p65-mediated inflammation ↓	[136]
Apelin-13	Cisplatin-induced AKI mice	0.1 μg/kg/day, i.p.	Serum CRE and BUN ↓, urinary NGAL and Kim-1 ↓	Mitochondrial profiles ↑, renal ATP ↑, PGC-1α/ERRα/SIRT3 signaling ↑	[137]
Apelin-13	Cyclosporine-induced AKI rats	15 μg/kg/day, s.c.	Tubulo-interstitial injury ↓	NO ↑, NFATc1 pathway ↑	[138]
Apelin-13	LPS-induced AKI rats	10 μg/kg/day, i.p.	Serum CRE and BUN ↓, renal damage ↓	Mitochondrial quality ↑, intrarenal RAS ↓	[139]
ELA-11, ELA-32	Mice with renal I/R injury	1.2 μmol/kg/day, s.c.	Renal fibrosis ↓, inflammation ↓, apoptosis ↓, DNA damage ↓, renal tubular lesions ↓, renal dysfunction ↓	-	[17]
PEGylated and Acylated ELA-11	Mice with renal I/R injury	300 pmol/kg/day, i.p.	Plasma stability ↑, APJ-binding capacity ↑, kidney injury ↓	-	[140]
FC-ELA-21	LPS-induced AKI mice	1 mg/kg/day, s.c.	Kidney injury ↓, cell apoptosis ↓, inflammation ↓, M1 macrophages ↓	PI3K/Akt signaling ↑	[141]
FC-ELA-21	Mice with renal I/R injury	1 mg/kg/day, s.c.	Kidney injury ↓, ROS ↓, cell apoptosis ↓	PI3K/Akt signaling ↑	[142]
ELA-32	Apela^Ksp^ KO mice with renal I/R injury	300 pmol/kg/day, s.c.	Pathological injury ↓, renal microvascular blood flow ↑	ARG2 ↓, PGE2 ↑	[104]
**Chronic kidney disease**					
Apelin-13	Mice with UUO	0.1 μmol/kg/day, i.p.	Renal interstitial fibrosis ↓	Tubular EMT ↓, TGF-β1/SMAD signaling ↓	[143]
Apelin-13	Mice with UUO	150 μg/kg/day, i.p.	Renal interstitial fibrosis ↓, myofibroblast accumulation ↓, interstitial macrophages ↓	APJ/Akt/eNOS signaling ↑	[144]
ELA-13	Mice with UUO	1.2 μmol/kg/day, s.c.	Renal fibrosis ↓, Renal function ↑	SMAD and ERK signaling ↓	[145]
[Pyr^1^]apelin-13	Patients with CKD	1 or 30 nmol/min for 30 min, i.v.	MAP↓, cardiac index ↓, systemic vascular resistance ↓, renal blood flow ↓, natriuresis ↓, proteinuria ↓, free water clearance ↓, GFR ↓, filtration fraction ↓	-	[15]

↓ represents inhibition or downregulation, ↑ represents activation or upregulation.

**Table 6:** Effects of exogenous Apelins or ELAs on diabetic nephropathy.

Peptides/Analogs/Antagonists	Experimental Models	Dose and Route	Outcomes	Associated Molecular Mechanism	Refs
Apelin-13	KkAy mice (diabetic mice)	30 μg/kg/day, i.p.	Renal dysfunction ↑, foot process injuries ↑	Podocyte apoptosis ↑, podocyte autophagy ↓	[166]
Apelin-13	KkAy mice (diabetic mice)	30 μg/kg/day, i.p.	Albuminuria ↑, foot process injuries ↑	Endoplasmic reticulum stress ↑, proteasome activities ↓	[167]
Apelin-77	STZ-induced diabetic mice	500 μg/kg/day, s.c.i	Renal fibrosis ↓	TGFβ/SMAD/CEBPA signaling ↓, EndMT ↓	[151]
Apelin-13	STZ-induced diabetic mice	30 μg/kg/day, i.p.	Glomerular fibrosis ↓, Glomerular basement membrane thickening ↓	Laminin ↓, collagen IV ↓, SIRT3/KLF15 signaling ↑	[168]
Apelin-13	KkAy mice (diabetic mice)	30 μg/kg/day, i.p.	Glomerular fibrosis ↓, EndMT ↓	BKCa/SP1/DKK1 signaling ↑, Wnt/β-Catenin signaling ↓	[169]
Apelin-13	KkAy mice (diabetic mice)	30 μg/kg/day, i.p.	Epithelial–mesenchymal transition ↓, glomerular fibrosis ↓	β5i/TGF-β1/SMAD signaling ↓	[170]
[Pyr^1^]apelin-13	FVB/Ove26 mice (diabetic mice)	150 μg/kg/day, s.c.	Kidney and glomerular hypertrophy ↓, renal inflammation ↓, albuminuria ↓	Antioxidant catalase ↑	[171]
Apelin-13	STZ-induced diabetic mice	40 or 400 pmol/kg/day, i.p.	Urine protein ↓, insulin ↑, insulin resistance ↑, renal fibrosis ↓	NO ↑	[172]
Apelin-13	Akita mice (diabetic mice)	400 pmol/kg/day, t.v.i.	GFR ↓, proteinuria ↓, glomerular hypertrophy ↓, mesangial expansion ↓, renal inflammation ↓	Histone hyperacetylation ↓	[173]
ELA-32	STZ-induced diabetic mice	4.5 mg/kg/day, s.c.	Renal inflammation ↓, kidney fibrosis ↓	PI3K/Akt/mTOR signaling ↑	[175]
ELA-21	db/db mice	5 mg/kg/day, i.p.	Kidney fibrosis ↓, renal damage ↓	Renal tubular autophagy ↑	[174]
Lentivirus expressing ELA	db/db mice	Intrarenal injection	CRE and BUN ↓, urinary albumin excretion ↓, glomerular endothelial injury ↓	AMPK/NLRP3 signaling ↑	[66]

↓ represents inhibition or downregulation, ↑ represents activation or upregulation.

**Table 7:** Effects of exogenous Apelins or ELAs on hypertensive kidney injury and cardiorenal syndrome.

Peptides /Analogs	Experimental Models	Dose and Route	Outcomes	Associated Molecular Mechanism	Refs.
**Hypertensive kidney injury**					
[Pyr^1^]apelin-13	L-NAME-treated mice (endothelial damage)	296 μg/kg/day, i.p.	Blood pressure (transiently) ↑	-	[181]
Apelin-13	ADMA-treated rats (endothelial damage)	10 nmol/kg/day, i.v.	Blood pressure ↑	MLC phosphorylation in VSMCs ↑	[182]
Apelin-13	Two-kidney-one-clip hypertensive rats	20 or 40 μg/kg, i.v	Blood pressure ↓	Gi and PKC signaling ↑	[183,184,185]
[Pyr^1^]apelin-13	Spontaneously hypertensive rats	2, 4, and 10 nmol/kg, i.v.	Blood pressure ↓	Phosphorylation of eNOS ↑	[187]
Apelin-13	Spontaneously hypertensive rats	24 nmol/day, minipump (PVN Microinjection)	Blood pressure ↑, cardiac hypertrophy ↑,	Sympathetic activation ↑, AVP release ↑	[11]
Apelin-13	Spontaneously hypertensive rats	3, 30, or 300 pmol, minipump (PVN Microinjection)	sympathetic outflow ↑, blood pressure ↑	Activating APJ receptor	[12]
Apelin-13	L-NAME-treated mice (endothelial damage)	60 nmol/kg/day, s.c.	MAP↓, kidney injury ↓	-	[189]
ELA-32	High salt-loaded Dahl salt-sensitive rats	1.5 mg/kg/day, minipump (s.c.)	Blood pressure ↓, kidney injury ↓, renal inflammation ↓, renal fibrosis ↓	Intrarenal RAS ↓	[16]
ELA gene delivery by AAV9	High salt-loaded Dahl salt-sensitive rats	i.v.	Blood pressure ↓, Renal fibrosis ↓	-	[106]
pcDNA3.1-ELA-32	DOCA/salt-treated rats	Intrarenal injection	Blood pressure ↓, Renal fibrosis ↓, renal inflammation ↓, kidney injury ↓	NADPH oxidase/ROS/NLRP3 signaling ↓	[190]
ELA-21	Spontaneously hypertensive rats	6 nmol/day, minipump (s.c.)	Diastolic blood pressure ↓, vascular inflammation ↓, oxidative stress ↓, VSMC proliferation ↓	PI3K/Akt signaling ↓, Nrf2 ↑	[59]
ELA-21	Spontaneously hypertensive rats	5.28 nmol/day, minipump (PVN Microinjection)	Blood pressure ↑, renal sympathetic nerve activity ↑, AVP release ↑	PI3K/Akt signaling ↑	[39]
ELA-21	Spontaneously hypertensive rats	5.28 nmol/day, i.v.	Blood pressure ↓, renal sympathetic nerve activity ↓	PI3K/Akt signaling ↓	[39]
ELA-32	Spontaneously hypertensive rats receiving high-salt diet	10 nmol/kg/hour, minipump (s.c.)	Blood pressure ↓, cardiovascular and renal dysfunctions ↓, Fibrosis ↓, hypertrophy ↓	Heart ACE ↓, ACE2 ↑, Neprilysin ↑; Renal ACE ↓, ACE2 ↓, Neprilysin ↕;	[191]
**Cardiorenal syndrome**					
[Pyr^1^]apelin-13	α-actin transgenic mice (cardiomyopathy model)	2 mg/kg/day, minipump (s.c.)	cardiac systolic impairment ↓, cardiac ventricular dilation ↓, diastolic dysfunction ↓	Peripheral vascular resistance ↓	[192]
Apelin-13	Rats with myocardial infarction-induced heart failure	10 nmol/kg/day, i.p.	Cardiac fibrosis ↓, oxidative stress ↓	PI3K/Akt signaling ↓	[193]
Apelin-13	Rabbits with sodium pentobarbital-induced acute heart failure	10 μg/kg/hour, minipump (i.v.)	Cardiac dysfunction and injury ↓, cell apoptosis ↓	APJ/Akt signaling ↑, Endoplasmic reticulum stress ↓	[194]
ELA-32	Mice with Type I cardiorenal syndrome (Acute myocardial infarction)	1 mg/kg/day, minipump (s.c.)	Cardiorenal function ↑, cardio-renal pathology ↓, systemic and renal inflammation ↓	APJ signalling ↑, NF-κB signalling ↓	[195,196]
ELA-32	Rats with cecal ligation puncture-induced peritonitis	39 µg/kg/hour, minipump (i.v.)	Myocardial dysfunction ↓, survival ↑, left ventricular filling ↑, plasma volume ↑, kidney injury ↓, free-water clearance ↓	Crosstalk with the vasopressinergic system	[197]

↓ represents inhibition or downregulation, ↑ represents activation or upregulation.

## Data Availability

Data sharing not applicable to this article as no datasets were generated or analyzed during the current study.
